# Supplementary figures and images for: Racing against change: understanding dispersal and persistence to improve species' conservation prospects
Source: Proc Biol Sci. 2020 Nov 25;287(1939):20202061. doi: 10.1098/rspb.2020.2061 (PMC7739496; doi:10.1098/rspb.2020.2061)

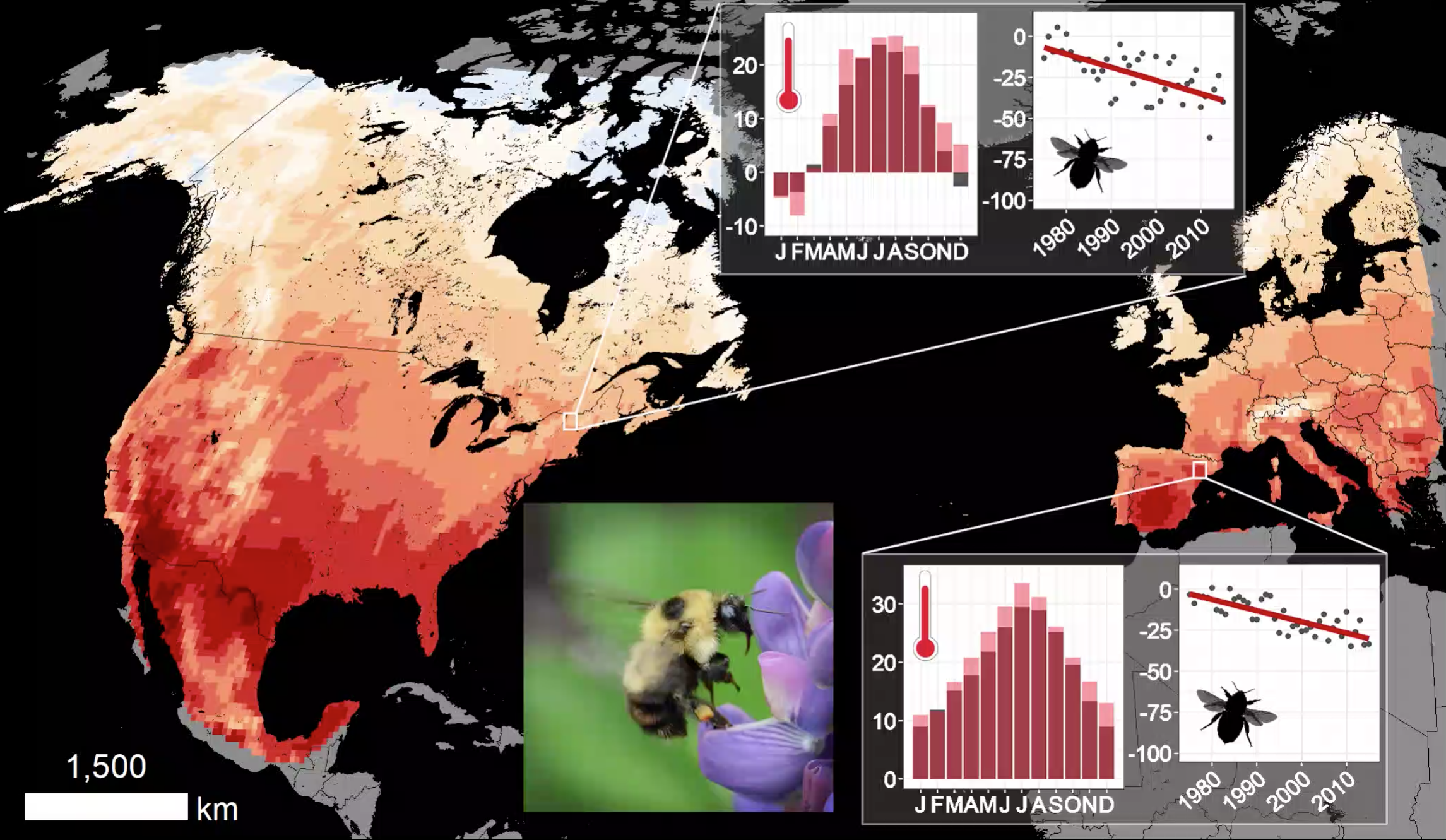

Supplement: Figure S1 [file rspb20202061supp1.tiff]
